# Insights on the mechanism of action of immunostimulants in relation to their pharmacological potency. The effects of imidazoquinolines on TLR8

**DOI:** 10.1371/journal.pone.0178846

**Published:** 2017-06-05

**Authors:** Carlos Kubli-Garfias, Ricardo Vázquez-Ramírez, Cynthia Trejo-Muñoz, Arturo Berber

**Affiliations:** 1 Instituto de Investigaciones Biomédicas, Universidad Nacional Autónoma de México, Ciudad de México, México; 2 Escuela Superior de Medicina, Instituto Politécnico Nacional, Ciudad de México, México; 3 Fundación para el Avance de la Ciencia, Ciudad de México, México; Bioinformatics Institute, SINGAPORE

## Abstract

Imidazoquinolines are powerful immunostimulants (IMMS) that function through Toll-like receptors, particularly TLR7 and TLR8. In addition to enhancing the immune response, IMMS also function as antineoplastic drugs and vaccine adjuvants. These small compounds display almost the same molecular structure, except in some cases in which atom in position 1 varies and changes the imidazole characteristics. A variable acyclic side chain is also always attached at atom in position 2, while another chain may be attached at atom in position 1. These structural differences alter immune responses, such as the production of interferon regulatory factor and nuclear factor-κB (IRF-NFκB). In this work, quantum mechanics theory and computational chemistry methods were applied to study the physicochemical properties of the crystal binding site of TLR8 complexed with the following six IMMS molecules: Hybrid-2, XG1-236, DS802, CL075, CL097 and R848 (resiquimod). The PDB IDs of the crystals were: 4R6A, 4QC0, 4QBZ, 3W3K, 3W3J, and 3W3N respectively. Thus, were calculated, the total energy, solvation energy, interaction energy (instead of free energy) of the system and interaction energy of the polar region of the IMMS. Additionally, the dipole moment, electrostatic potential, polar surface, atomic charges, hydrogen bonds, and polar and hydrophobic interactions, among others, were assessed. Together, these properties revealed important differences among the six TLR8-immunostimulant complexes, reflected as different interaction energies and therefore different electrostatic environments and binding energies. Remarkably, the interaction energy of a defined polar region composed of the highly polarized N3, N5 atoms and the N11 amino group, acted as a polar pharmacophore that correlates directly with the reported immunopharmacological potency of the six complexed molecules. Based on these results, it was concluded that accurate physicochemical analysis of the crystal binding site could reveal the binding energy (measured as interaction energy) and associated molecular mechanism of action between IMMS and TLR8. These findings may facilitate the development and design of improved small molecules with IMMS properties that are targeted to the TLR system and have enhanced pharmacological effectiveness and reduced toxicity.

## Introduction

The discovery of interferon by Isaacs and Linderman [1] was a milestone in immunology. This cytokine is produced and released in response to the harmful effect of viruses, bacteria, and parasites, as well as other strange or invasive cells. However, interferon was not purified and thus available for therapy and research until 20 years later [2]. Consequently, those findings prompted the search for modulators of the immune system that are capable of inducing interferon synthesis. Interestingly, nucleosides appeared to be good candidates to activate interferon production and, accordingly, the immune system. Notably, an early work utilizing pyrimidine derivatives indicated a clear antibacterial activity of, particularly, thymine and purine compounds [3].

Several antiviral compounds derived from nucleosides have been developed to activate the immune response. Among the original modified nucleosides that initiated modern immunopharmacology were the following: the nucleoside iododeoxyuridine [4], the tri-fluorinated uracil nucleoside Trifluridine [5] and the iodinated compound 5-iodo-2-deoxyuridine named Vidarabine [6]. The guanosine derivative hydroxyethoxymethyl-guanine, (acyclovir) synthetized by Elion *et al*. in 1977 [7] may be the best example of a successful antiviral agent based on a purine.

Based on the adenine molecule, imidazoquinoline (IMZQ) compounds were developed also with the aim of fighting viral infections. In addition to the adenine molecule, the structure of IMZQ includes a third ring to yield the quinoline moiety joined to the five-membered aromatic heterocyclic imidazole ring. Among the first synthetized IMZQ was the compound S-25059, followed by imiquimod (S-26308, R837) in 1983 [8]. The analog resiquimod (S-28463, R848) was later synthetized and released, as well as antiviral compound [9]. The induction of interferon by imiquimod was demonstrated in early studies [10], and consequently both imiquimod and resiquimod have been typified as immunomodulators [11]. It is currently accepted that IMZQ is capable of modifying the immune response to induce antiviral and antitumor activity, basically via cytokine production, e.g., interferon-α induction, mediated partially by NF-κB activation, which in turn stimulates the innate immune response and acquired immunity [8, 11].

However, an important issue is the activation of the immune response by Toll like receptors (TLRs) induced by IMZQ, particularly TLR7 and TLR8, which function in immune responses against viral infections [12]. Thus, the structural basis of the mechanism of action of IMZQ and, therefore, IMMS has been unveiled in a larger capacity by valuable studies applying X-ray crystallography. In this regard, several IMMS analogs have been crystallized, showing binding particularly to TLR8 and revealing that IMMS form a bridge between two TLR molecules. Based on these findings, we selected six IMMS complexed with TLR8: Hybrid-2 [13], XG1-236, DS802 [14], CL075, CL097, and R848 [15]. The aim was to explore the binding energy calculated as interaction energy, along with binding characteristics associated with their pharmacological activity. To achieve this goal, we studied the binding core of the six IMMS-TLR8 complexes from the reported X-ray structures. These cores were studied comparatively based on their electronic structure and molecular properties. For comparison, two unbound IMMS antagonists (compounds 15 and 16) reported by Shukla *et al*. [16] were also examined to characterize their molecular and electronic properties.

## Methods

### IMMS agonists and antagonists

We evaluated and compared the molecular properties of crystal structures of the following IMMS agonists: **Hybrid-2** (1-(4-amino-2-butyl-1H-imidazo [4, 5-c] quinolin-1-yl)-2-methylpropan-2-ol), **XG1-236** (2-butyl-2H-pyrazolo [3, 4-c] quinolin-4-amine), **DS802** (2-butyl [1, 3] oxazolo [4, 5-c] quinolin-4-amine), **CL075** (2-propyl [1, 3] thiazolo [4, 5-c] quinolin-4-amine), **CL097** (2-(ethoxymethyl)-1H-imidazo [4, 5-c] quinolin-4-amine), and **R848** (1-[4-amino-2-(ethoxymethyl)-1H-imidazo [4, 5-c] quinolin-1-yl]-2-methylpropan-2-ol). All of them were complexed with TLR8 and retrieved from the Protein Data Bank (PDB ID: 4R6A, 4QC0, 4QBZ, 3W3K, 3W3J, and 3W3N respectively) [17]. The IMMS-TLR8 complexes have a dimeric form (two TLR monomers; A and B) and two ligands. Each ligand was extracted from the receptor, and its total energy was calculated, allowing only the lowest energy IMMS monomer for the whole physicochemical study. Likewise, the root mean square deviation (RMSD) for each pair of the same IMMS molecules, were measured using the VMD program [18]. Additionally, two IMMS antagonists were assessed: **compound 15** (1-(4-amino-2-((ethylamino) methyl)-3H-imidazo [4, 5-c] quinolin-3-yl)-2-methylpropan-2-ol) and **compound 16** (2-((ethylamino) methyl)-3H-imidazo [4, 5-c] quinolin-3-yl)-2-methylpropan-2-ol) [16]. The crystal structure of compound 16, was taken from the Cambridge Crystallographic Data Centre (Accession number: CDC 718787) [19] and was used to model compound 15 by adding an amino group at C4. The added amino group at position 11 was locally optimized with the Density Functional Theory (DFT) using the ωB97X-D function and the 6-31G* basis set level with constraints in the other atoms to prevent changes in the crystal conformation of the new molecule. Finally, the energies and physicochemical properties of agonists and antagonists were evaluated in a single point calculation with DFT using the ωB97X-D function and the 6-31G* basis set level. The star (*) indicates that the basis was polarized. The studied molecular properties were as follows: total energy, solvation energy, highest occupied molecular orbital (HOMO) and lowest unoccupied molecular orbital (LUMO) energies, hardness, electronegativity, polarizability, electrostatic charges, dipole moment (DM), polar surface area, electrostatic potential (EP), molecular area and volume.

### Interaction analysis of the IMMS-TLR8 complex

The interactions between the crystal structures of Hybrid-2, XG1-236, DS802, CL075, CL097 and R848 complexed to TLR8 were analyzed. Only the first core of residues belonging to the TLR8 binding pocket was included, and the cut off distance between the IMMS and the receptor was set at 4 Å. The included amino acids ranged from 9 to 12, among all TLR8-IMMS complexes. Residues showing hydrophilic (polar) interactions achieved a distance of approximately 3.3 Å, while those with hydrophobic interactions reached 4 Å with respect to the IMMS atoms. The Ligand Explorer program was used for this assessment. The geometrical parameters (Å) were set up as follows: hydrogen bonds: 3.3, water bridged hydrogen bonds: 3.3, and hydrophobic interactions: 4.0 [20]. Thus, important binding interactions were studied, namely: hydrogen bonds, water bridged hydrogen bonds, hydrophobic interactions (van der Waals) and electrostatic charges of atoms of the agonists interacting with those atoms in the receptor amino acids. The number and type of molecular interactions of the TLR8-agonist complex and the calculation of the interaction energy of each complex permitted the evaluation of the energy of the receptor-ligand interaction, which was considered an index of the extent of binding.

**Fig 1:**
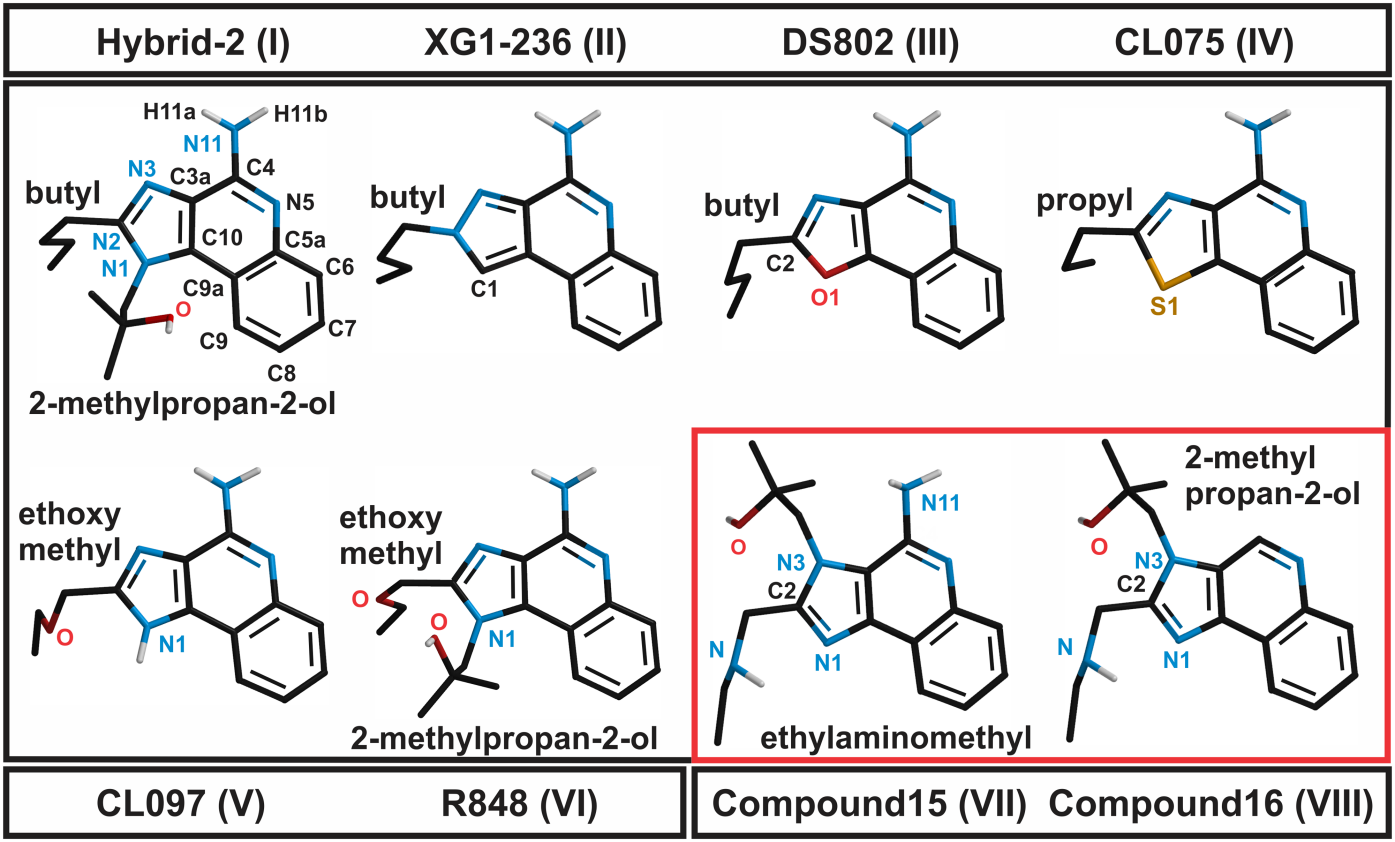
Molecular structure and numbering of the studied IMMS. Compounds are arranged from the highest to the lowest pharmacological potency. The proposed polar pharmacophore of the agonists is formed by N3 and N5 atoms, the N11 amino group and the C4 atom. The variety of atom at position 1 and side chains are indicated. The antagonist compounds 15 and 16 differ in the polar pharmacophore, possessing an N3 side chain, and compound 16 lacks the N11 amino group.

Two interaction energy values were calculated; the total interaction energies included the IMMS molecule, all adjacent binding pocket residues and molecules of water (hydrophilic and hydrophobic interactions) and the selected polar region interaction energy, that included N3, N5 and the N11 amino group as well as the C4 atom of the IMMS compound (this region was defined as: the polar pharmacophore), whereas from the TLR side, were included two residues Asp543 and Thr574, as well as, a fixed molecule of water. Fig 1 displays the atom numbers and positions of agonist and antagonist IMMS.

A simplified procedure to calculate the interaction energy is provided by the following formula:

IE=[RLW]−[R+L+W],

where **IE** is the interaction energy of binding, **RLW** is the energy of the complex, formed by the receptor amino acids, the ligand and water energies, **R** is the energy of the amino acids of TLR8, **L** is the energy of the ligand agonist, and **W** is the energy of molecules of water.

The binding interaction energies were determined by single point calculations using the DFT with the ωB97X-D function and the 6-31G* basis set level. Likewise, the volume of the binding pocket cavities of each TLR8 dimer was calculated using the Swiss-Pdb Viewer program [21]. All ab initio calculations were conducted using Spartan'14 software [22]. The computer consisted of an eight-core AMD processor at 4.0 GHz. A line regression and correlation statistical analysis, comparing the natural logarithm values of TLR8 agonist activity and pharmacophore interaction energy values was calculated with Origin 8 Pro (OriginLab, Northampton, MA, USA).

## Results

The studied agonist molecules were as follows: Hybrid-2 (I) [13], XG1-236 (II), DS802 (III) [14], CL075 (IV), CL097 (V) and R848 (VI) [15]. The antagonists were compound 15 (VII) and compound 16 (VIII) [16]. Roman numerals were added to identify easily the studied IMMS. All compounds had three rings forming a planar system that included the imidazolquinoline moiety. The C4 atom, the N3 and N5 atoms and the amino group of N11 may be considered as a polar pharmacophore.

The atom at position 1 may be either C (XG1-236), O (DS802), or S (CL075). Likewise, an amino group (CL097) or a 2-methylpropan-2-ol side chain may be found at position 1 (Hybrid-2 and R848). At position 2, diverse side chains are found, namely butyl (I and III), N2-butyl (II), propyl (IV) and ethoxy methyl (V and VI). The antagonist molecules 15 and 16 have a nitrogen atom at position 1, an ethylaminomethyl side chain at position 2, and a 2-methylpropan-2-ol side chain at N3. Clearly, the structure of the antagonist modifies importantly the polar pharmacophore region because of the steric bulk of the N3 side chain in both antagonist molecules and the absence of the N11 amino group in compound 16. The potency of the IMMS agonists as well as the antagonistic IMMS seem to be related to the chemical structure and associated physicochemical properties. Fig 1 displays the chemical structures of both agonist and antagonist IMMS.

### Immunostimulant potency of IRF-NFκB transcription factors

The studied IMMS show different extents of IRF-NFκB transcription factor stimulation of TLR7 and TLR8. Thus, the EC_50_ data show different pharmacological potency for TLR7 and TLR8 in the same IMMS. However, the pharmacological potency of the agonists Hybrid-2 [13] and XG1-236 [14] acts first and second in both TLR7 and TLR8, respectively.

To stimulate IRF-NFκB, XG1-236 [14], DS802 [14] and CL075 [23] are more effective for TLR8, whereas Hybrid-2 [13], CL097 [24] and R848 [25] are more effective for TLR7. The included antagonist compounds 15 and 16 [16] exert their antagonistic effect with higher and lower EC_50_ doses, respectively. Table 1 shows the pharmacological potencies of TLR7 and TLR8 agonists. The EC_50_ of antagonist compounds 15 and 16 are also included.

**Table 1:** Potencies of IMMS (EC_50_) for IRF-NFκB stimulation in TLR7 and TLR8.

Agonist	EC_50_ (μM) (TLR8)	Potency (TLR8)	EC_50_ (μM) (TLR7)	Potency (TLR7)	Reference
Hybrid-2	0.019	1°	0.0025	1°	[13]
XG1-236	0.056	2°	0.190	2°	[14]
DS802	0.180	3°	0.550	4°	[14]
CL075	0.400	4°	4.000	6°	[23]
CL097	4.000	5°	0.400	3°	[24]
R848	6.400	6°	1.400	5°	[25]
**Antagonist**					
Compound 15	---	---	25.0	2°	[16]
Compound 16	---	---	7.5	1°	[16]

### IMMS crystal structures

**Fig 2:**
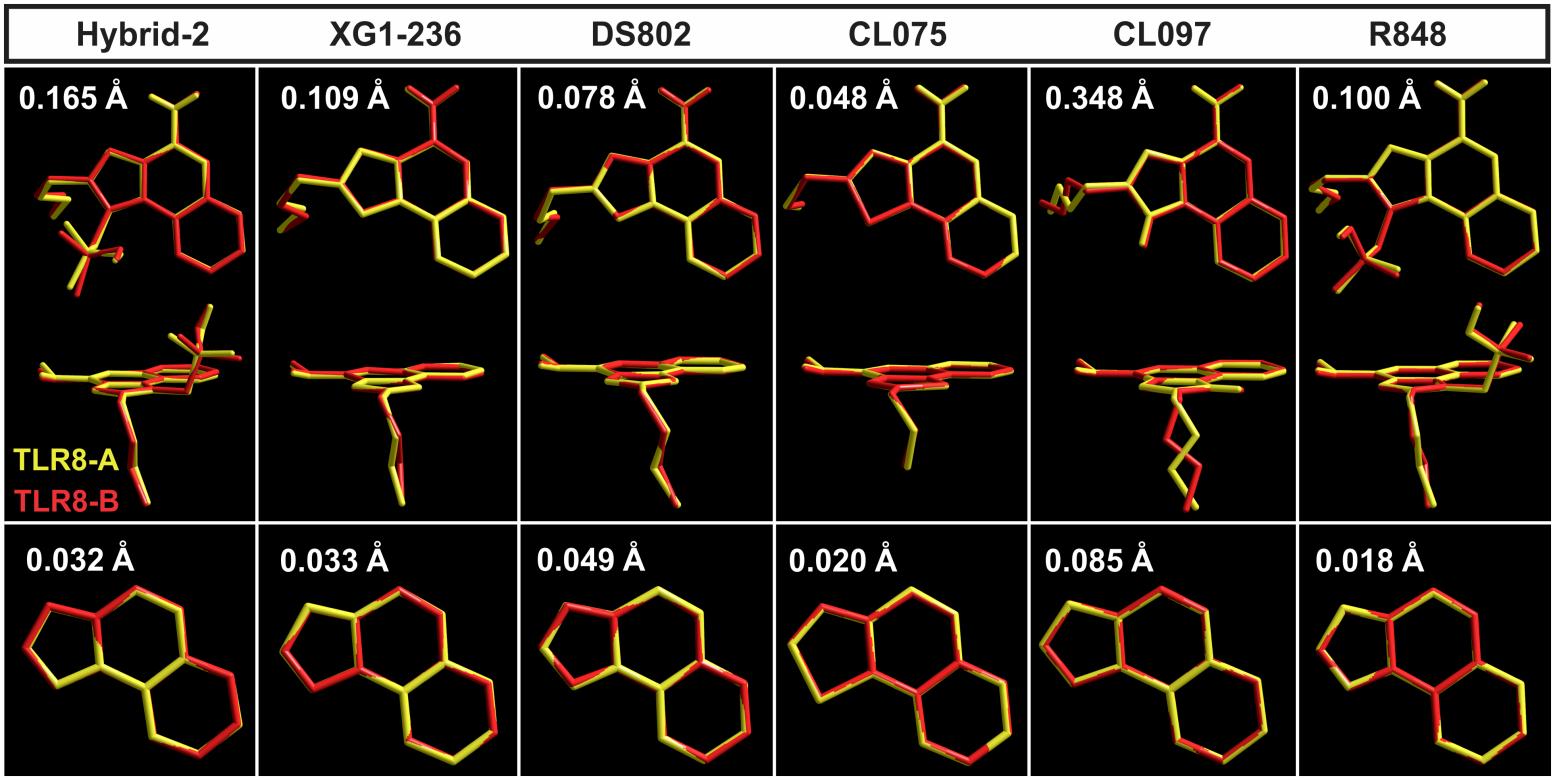
RMSD values and superimposed views of monomers A and B of the IMMS agonist. Superimposition of compound CL097 showing the interlaced 2-ethoxymethyl side chain that markedly increases the RMSD value. The main differences are due to the side chains because the five-sided ring system alone shows significantly lower values.

The six IMMS agonists isolated from TLR8 crystals have two monomers, namely, A and B. The pairs of monomers for each agonist were compared by measuring the root mean square deviation (RMSD) of the atomic positions. The largest RMSD difference was found in the CL097-A and B pair due to the 2-ethoxymethyl side chain, which adopts two opposite conformations that interlace when superimposed. Fig 2 shows the IMMS superimposition and RMSD values.

### Assessment of the physicochemical properties of IMMS

The molecular and physicochemical properties of both A and B crystal monomers were calculated by single point ab initio calculations. The monomers with the highest energy were discarded, leaving for further analysis only the monomers with the lowest energy. The physicochemical values of both agonists and antagonists show some differences due to the varied substitution of atoms and inherent side chains. The properties hardness (η) and electronegativity (χ) were calculated as follows: *η* = (*εLUMO* − *εHOMO*) / 2 and *χ* = − (*εLUMO* + *εHOMO*) / 2 respectively.

Both antagonists were studied; however, the crystal of antagonist compound 15 was not available. Therefore, compound 15 was modeled using the crystal of compound 16 as a model and adding an amino group at position 11. Table 2 shows the values for the electronic and physicochemical properties of the lowest energy IMMS conformers from single point ab initio calculations. The molecular properties of the antagonists are also included.

**Table 2:** Values of the molecular properties of monomers of IMMS crystals from TLR8 complexes.

Agonist	Total Energy (a.u.)	Solvation Energy (kJ/mol)	HOMO (eV)	LUMO (eV)	Hardness(η)	Elect.(χ)	Polarizability (Å^3^)	Dipole (Debyes)	Polar Surface (Å^2^)	Area (Å^2^)	Volume (Å^3^)
Hybrid-2-B*	-994.289	-67.52	-6.91	1.51	4.21	2.70	66.30	6.29	57.42	342.27	331.76
XG1-236-B*	-761.868	-61.47	-6.98	1.03	4.01	2.98	60.10	4.13	41.74	272.90	254.16
DS802-A*	-781.749	-51.03	-7.26	0.83	4.05	3.22	59.80	2.58	47.34	271.71	250.68
CL075-B*	-1065.421	-45.09	-7.60	0.17	3.89	3.72	59.24	2.93	40.12	258.92	242.82
CL097-A*	-797.782	-67.13	-7.13	1.03	4.08	3.05	59.34	3.04	58.51	267.76	245.23
R848-A*	-1030.162	-62.97	-7.41	0.97	4.19	3.22	65.72	5.12	63.91	335.99	324.53
**Antagonist**											
Comp.15**	-1010.341	-56.80	-7.48	0.96	4.22	3.26	65.99	4.47	64.15	343.08	328.01
Comp.16**	-955.001	-51.42	-7.50	0.76	4.13	3.37	65.24	4.60	42.69	335.09	318.26

* Monomer with lowest energy.

** Compounds.

The three agonists with the lower total energy were compounds I, IV and VI because of the sulfur atom in compound IV and the large number of atoms in I, IV and VI. In addition to the lowest energy, CL075 (IV) also has the lowest area, volume, polarizability, polar surface, hardness, HOMO and LUMO, and the highest solvation energy and electronegativity. The molecular properties followed roughly three trends; one trend includes total energy, HOMO and LUMO excluding compound I. A second trend includes most of the following properties: area, volume, polar surface area, DM, polarizability and, to some extent, hardness. Finally, the third trend is consistent with the solvation energy and electronegativity.

**Fig 3:**
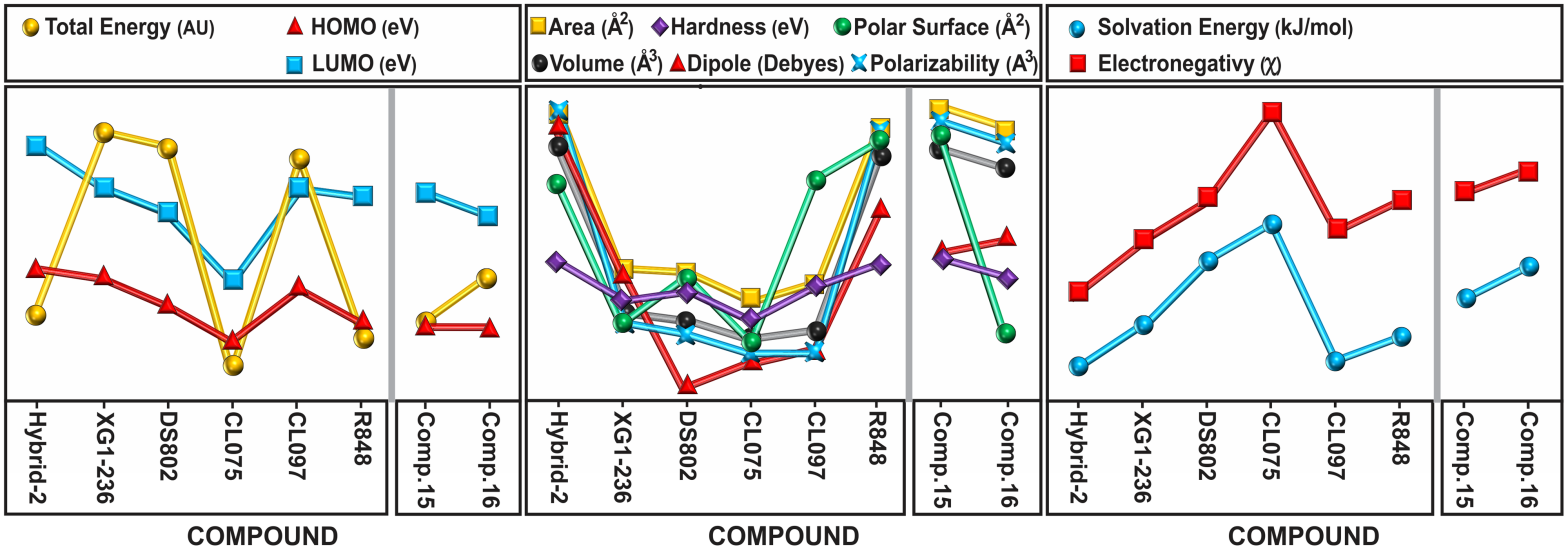
Superimposed curves depicting molecular properties with similar trends. Compounds are labeled I to VIII. The total energy tendency shows the lowest value for IMMS IV followed by VI and I. Clearly, the S atom in CL075 (IV) produces either the lowest or highest values for all properties excluding the DM. The similar structures of compounds I and VI produce marked property similarities. Because of the slight differences among the molecules and properties, the tendencies seem to correlate quite well in most cases. The antagonists (VII and VIII) show similar tendencies, in both cases somewhat excluding the total energy and polar surface. The properties of the curves are colored, and the compounds are marked to facilitate identification.

Interestingly, the compound 16 antagonist lacking the N11 amino group is more potent than its related compound 15. Coincidentally, compound 16 has 7 of the 11 physical and electronic properties with lower values: area, volume, polar surface, HOMO, LUMO, hardness and polarizability. Because of its large number of atoms, compound 15 has higher total and solvation energies, resulting in a larger DM and increased electronegativity. Fig 3 shows correlated curves of the IMMS molecular properties.

### Display and comparison of IMMS electrostatic potentials

All IMMS have a very similar EP in the polar pharmacophore region. Thus, atoms N3 and N5 show an intense negative EP and a marked positive EP in the N11 amino group. In contrast, the EP of the antagonists exhibited a different pattern, particularly compound 16, which lacks the N11 amino group. Indeed, the five-membered EP ring is the most versatile because of its atomic variety at position 1 and marked differences in the attached side chains. Molecules I and VI differ only in one carbon atom, in which an oxygen is interchanged in the side chain attached to atom at position 2. This feature produces an extra negative EP in VI and somewhat decreases the magnitude of the dipole moment and direction ranking for the second value as compared with compound I, which has the largest DM among the six IMMS. Additionally, molecules I and VI present the hydroxyl group of the side chain attached to atom at position 1 are in opposite position. These differences, however, are sufficient to situate these compounds as the most and less potent, respectively.

**Fig 4:**
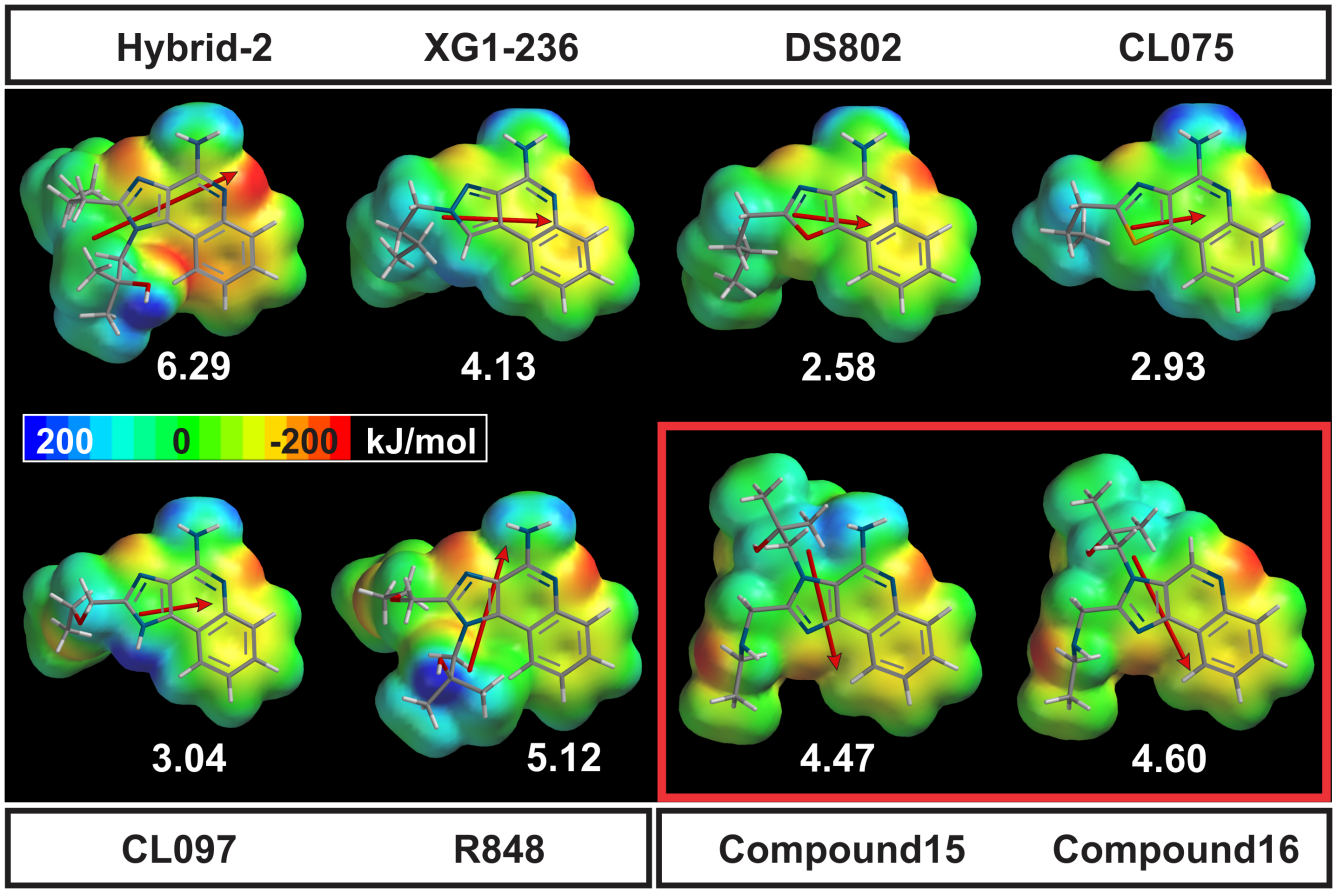
EP maps and DM vectors of agonist and antagonist IMMS. The EPs are encoded in the van der Waals volume, showing the almost similar patterns of the agonists in the polar pharmacophore region. The remaining part of the molecule varies according to the atoms occupying atomic position 1 and the different attached side chains (for atom numbering see Fig 1). The DM arrows cross the molecule from atom at position 2 to atom at position 5 (compounds II to IV) or point directly to the polar pharmacophore region with the largest values (compounds I and VI). Notably, the DM of the antagonists points opposite to the polar pharmacophore. The DM values are larger for antagonists favoring compound 16. The EP values were cut off at -200 and 200 kJ/mol.

Molecules II to V have a very similar EP pattern with a variable DM value but a similar direction. The EP of the antagonists are larger than those of the agonists and are also quite different in the polar pharmacophore region, also showing a yellow region in the benzen ring of the quinoline moiety, which indicates a tendency toward a negative charge. Notably, the antagonists have larger DM values and point opposite to agonist VI. Fig 4 shows both the EP and DM vector of the studied agonists and antagonist IMMS.

### Analysis of the crystal IMMS-TLR8 Interaction

**Fig 5:**
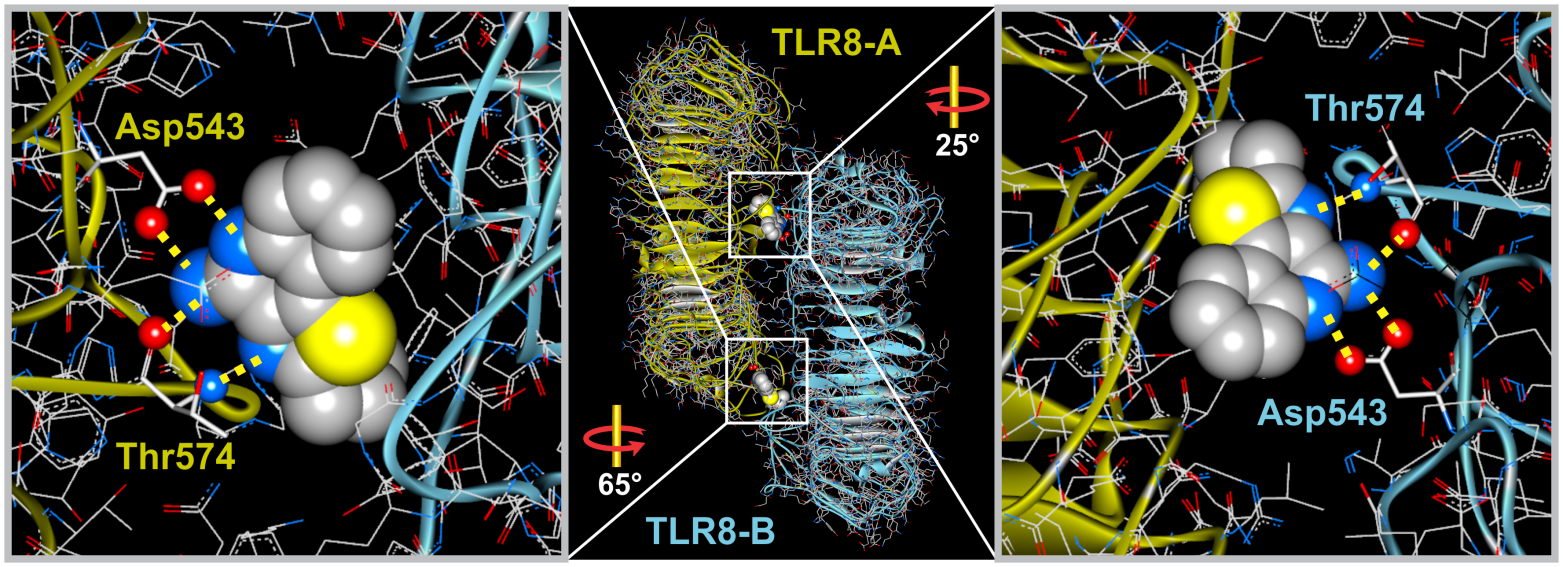
Example of the binding bridge between CL075 (IV) and the TLR8 dimer. The two TLR8 monomers are colored differently to show their relationship with the two CL075 ligands in between. The insets show a magnified view that includes the two ligand molecules bound to residues Asp543 and Thr574. The (blue) atoms N3 and N5 and the N11 amino group that make up the polar pharmacophore form in all cases hydrogen bonds with the oxygen atoms (red) of the two residues, (for atom numbering see Fig 1). The ligands clearly have an inverted position with respect to the two TLR8 monomers. Marked angles show the rotation differences between the ligands.

The crystals of the six IMMS-TLR8 complexes show similar binding patterns that include both A and B monomers of the receptor, forming a dimer bridged by two molecules of the ligand. Notably, one TLR8 monomer binds the ligand with polar residues, and the second monomer makes mainly hydrophobic contacts with the quinoline ring system and the side chains. For the second ligand molecule, the inverse occurs; therefore, the atomic arrangement and the residues involved are similar in both ligands. Fig 5 shows a typical example of the TLR8 dimer, including the region between the two molecules of CL075 (PDB ID: 3W3K).

### Binding pocket area and volume

**Fig 6:**
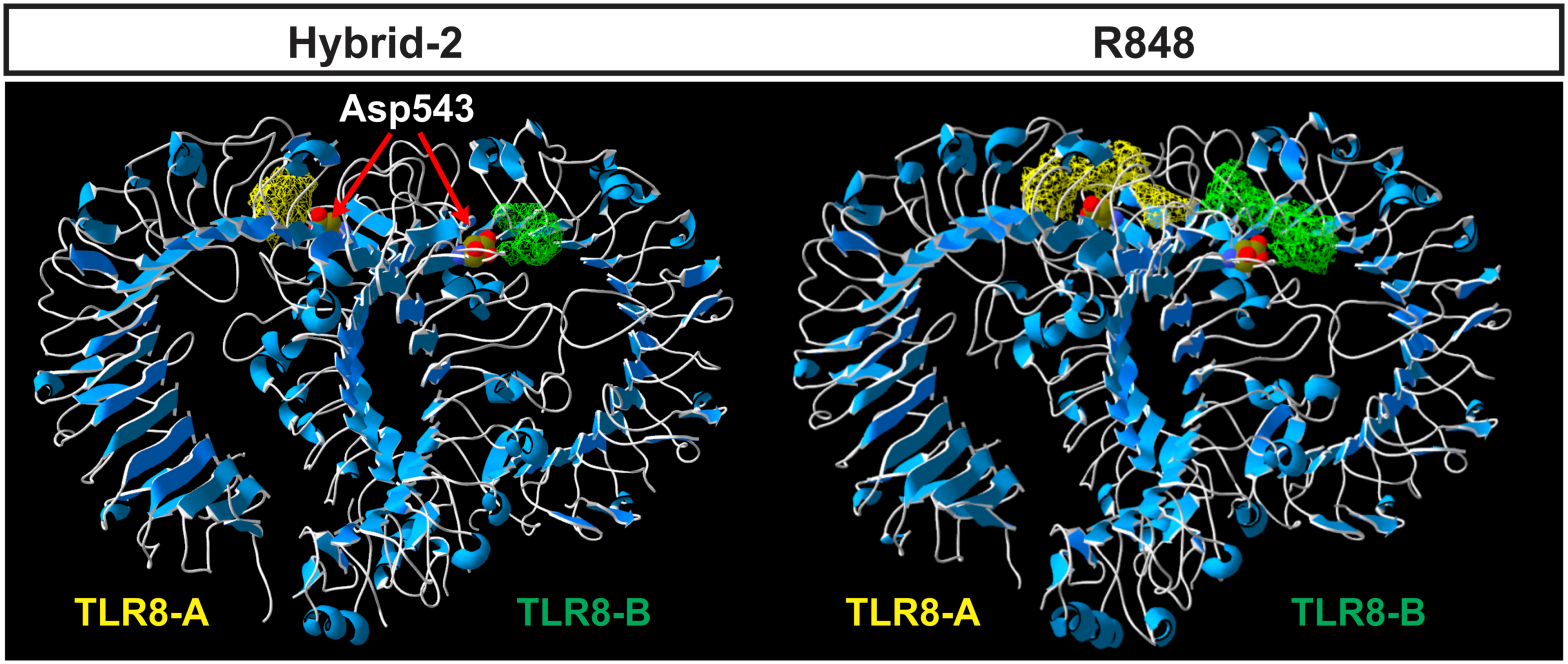
Binding pocket shape and position interconnecting the two TLR8 monomers of the IMMS molecules. Binding pockets allocating compounds I and VI are shown. The binding pocket of compound VI is clearly the largest. Residue Asp543 is shown as a reference.

Each of the two IMMS bound to the TLR8 dimer is enclosed in a cavity that forms the binding pocket. Thus, the volume of each binding pocket of the studied TLR8 agonists was calculated. Some cavities showed important differences among the six IMMS, particularly compounds IV and VI. However, the volume of the IMMS was quite similar among them. However, as expected, compounds I and VI had the highest volume because of its additional side chain. Two examples of the shape and position of the binding pocket for each TLR8 dimers are shown in Fig 6, and values of the binding pocket volume and IMMS physical properties are shown in Table 3.

**Table 3:** Volume of the binding pocket of TLR8 and agonists.

Cavity volume (Å^3^)	Hybrid-2	XG1-236	DS802	CL075	CL097	R848
TLR8-A	344.0	397.0	360.0	510.0	365.0	754.0
TLR8-B	399.0	378.0	343.0	580.0	363.0	671.0
**Agonist volume (Å**^**3**^**)**						
Monomer A	328.44	255.20	250.68	243.27	245.23	324.53
Monomer B	331.76	254.16	249.70	242.82	245.07	324.18

### The inner shell arrangement of the IMMS-TLR8 complex

The shell of the interacting residues in the binding pocket shared by the two monomers of the TLR8-IMMS complex at a distance of 4 Å, shows seven residues in common in the six studied agonists. Thus, in compounds I, II and IV four of them are in monomer A (Tyr348, Tyr353, Val378 and Phe405) while the remaining three (Asp543, Asp545 and Thr574) are in monomer B. In compounds III, V and VI the opposite occurs. Additionally, seven residues are found, to various extents, among the binding pockets bound to the IMMS: Phe346; Glycines 351, 376, and 572; Ser352; Arg429 and Val573. Thus, compounds II, IV and VI are bound to nine, compounds I and III to 11 and compound V, to 12 residues, respectively. Table 4 shows the distribution of residues by monomer for the studied TLR8 dimers.

**Table 4:** TLR8 residue distribution in both monomers that bind IMMS.

IMMS	Monomer	Residues	R*	Monomer	Residues	R*	Total R*
**Hybrid-2**	A	Tyr348, Tyr353, Val378, Phe405,	7	B	Asp543, Asp545, Thr574,	4	11
		**Phe346, Gly376, Arg429**			**Val573**		
**XG1-236**	A	Tyr348, Tyr353, Val378, Phe405,	6	B	Asp543, Asp545, Thr574	3	9
		**Gly376, Arg429**					
**DS802**	B	Tyr348, Tyr353, Val378, Phe405,	7	A	Asp543, Asp545, Thr574,	4	11
		**Phe346, Gly376, Arg429**			**Val573**		
**CL075**	A	Tyr348, Tyr353, Val378, Phe405,	6	B	Asp543, Asp545, Thr574	3	9
		**Gly351, Ser352**					
**CL097**	B	Tyr348, Tyr353, Val378, Phe405,	7	A	Asp543, Asp545, Thr574,	5	12
		**Phe346, Gly376, Arg429**			**Gly572, Val573**		
**R848**	B	Tyr348, Tyr353, Val378, Phe405,	6	A	Asp543, Asp545, Thr574	3	9
		**Phe346, Gly376**					

R* = number of residues.

The seven residues are similar in all compounds and shared between both monomers.

Dissimilar residues are in bold face.

**Fig 7:**
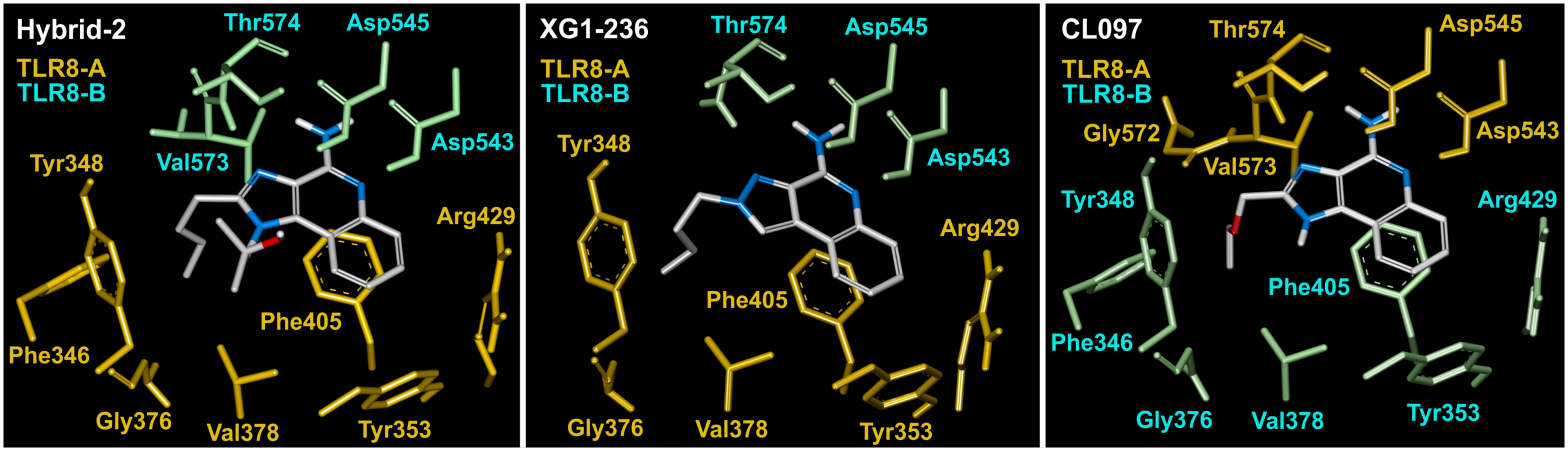
Polar and hydrophobic interactions among TLR8 and IMMS. The number of interacting residues varies somewhat among the six IMMS. The presented examples are Hybrid-2, XG1-236 and CL097 with 11, 9 and 12 interacting residues, respectively. Residues Asp543 and Thr574 are clearly close to the N atoms of the pharmacophore and belong to one TLR8 monomer, while the remaining residues, which elicit mostly hydrophobic interactions, belong to the TLR8 counterpart monomer.

In all the IMMS-TLR8 complexes, the two polar amino acids Asp543 and Thr574 interact with N3, N5 and N11 atoms of the polar pharmacophore. It is important to recall that one TLR8 monomer interacts with its polar residues binding the ligand polar pharmacophore, and the second TLR8 monomer interacts with the same ligand, mostly with hydrophobic residues through van der Waals forces. The second ligand interacts in the opposite manner, similarly forming a bridge between both TLR8 monomers. Fig 7 shows the interaction at a distance of 4 Å of three typical IMMS-TLR8 complexes. The residues are colored to be distinguished by their respective monomers.

### Electrostatic potential of the IMMS-TLR8 complex

The EP maps of the IMMS-TLR8 complex at a distance of 4 Å show important differences in the polar region, mainly due to the different number of fixed water molecules forming the hydrogen bonds network. Two EP patterns were observed; one pattern occurs in IMMS I, III and V, with two fixed water molecules and 11, 11 and 12 residues, respectively. A second pattern appears in IMMS II, IV and VI, with one, four and two fixed waters and nine residues in each. The first pattern includes Val573. The variety of atoms at position 1 somewhat modifies the EP pattern in neighboring atoms of the imidazole ring.

**Fig 8:**
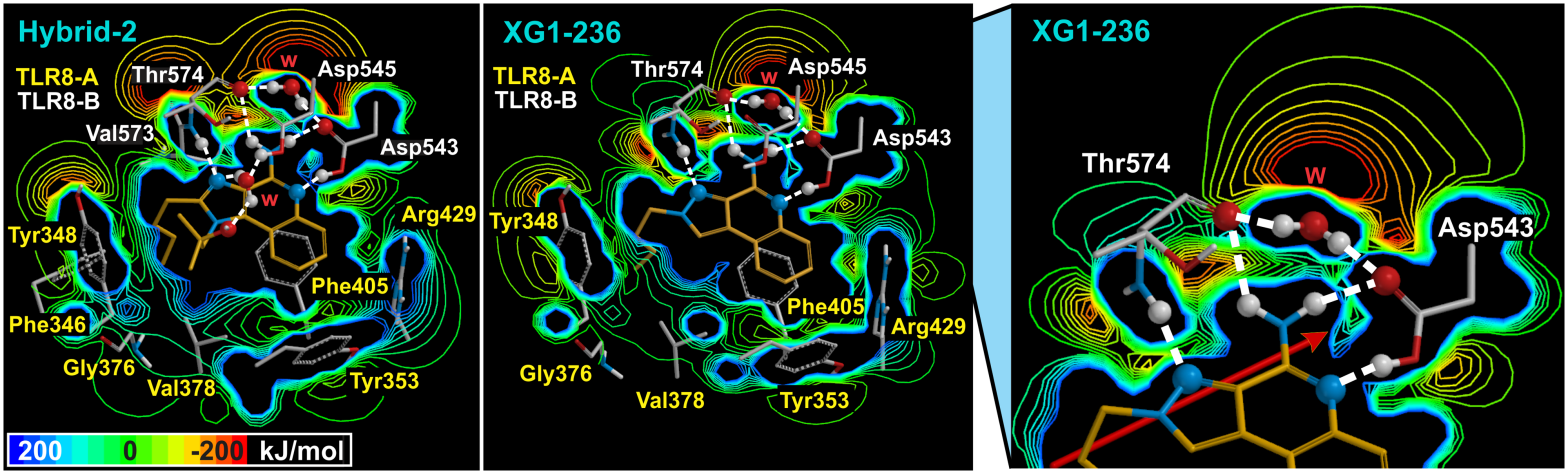
Slice-map views of the EP from the IMMS-TLR8 complex. Hybrid-2 (I) and XG1-236 (II), are shown for pattern one and pattern two, respectively. Likewise, an inset of the polar region of XG1-236 is shown. A clear definition of both polar and hydrophobic regions is observed, including the matching of anionic and cationic groups of some residues with the IMMS molecule. The inset shows a network of six hydrogen bonds. Four hydrogen bonds form a ring among a fixed molecule of water (W), Asp543 and Thr574 and the two hydrogen atoms of the N11 amino group. The same residues form two more hydrogen bonds to N5 and N3, respectively, (for atom numbering see Fig 1). EP energy was lost from -200 to 200 kJ/mol.

Similarities are observed for the different side chains of IMMS. Additionally, the hydrophobic interactions show important variations because of differences in the number of interacting residues. However, the most hydrophobic region (the quinolone-benzene ring) showed low variation in all cases. Notably, a neat and well defined highly polar IMMS pharmacophore region was identified, which included N3, N5 and the N11 amino group and the C4 atom. In that region, Asp545 and Thr574 form in all the studied IMMS-TLR8 dimer complexes a remarkable hydrogen bonds pattern with one fixed water molecule. Fig 8 shows EP slice views of both patterns of the IMMS-TLR8 dimer complex.

### Hydrophobic interactions of IMMS in the TLR8 binding pocket

**Fig 9:**
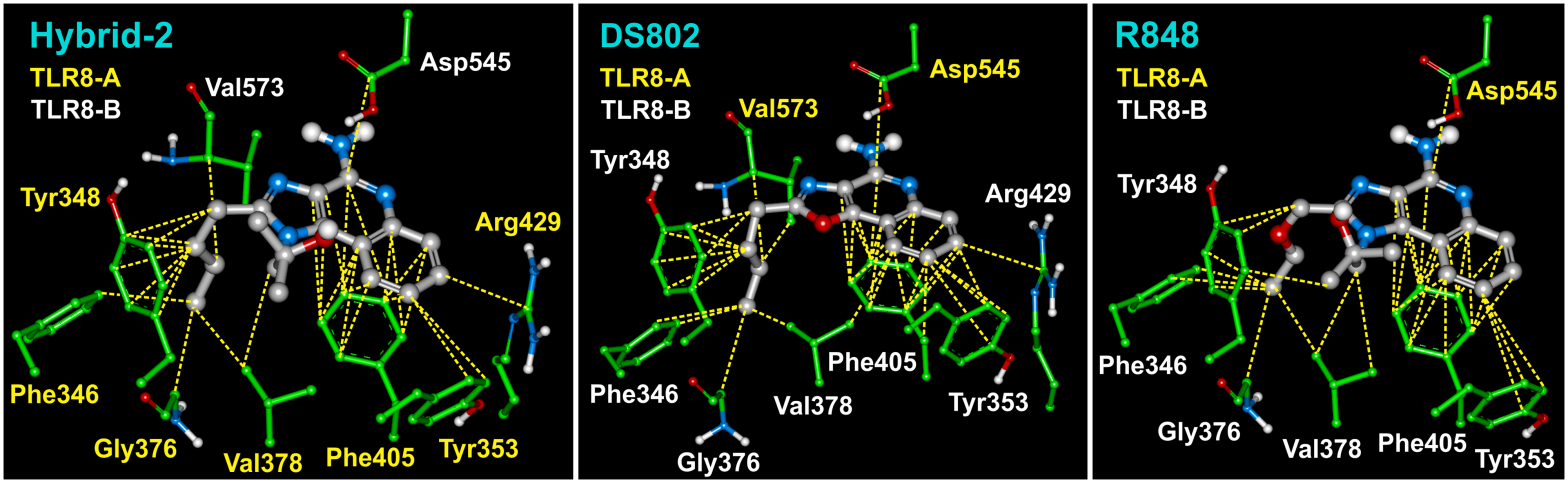
Three examples of hydrophobic interactions of IMMS with the TLR8-dimer complex. Compounds I, III and VI are shown having 9, 9 and 7 residues with 33, 37 and 32 hydrophobic interactions, respectively. In all cases, residues Tyr348, Tyr353, Val378, Phe405 and Asp545 are present. Asp545 belongs to the opposite TLR8 monomer. Residues Tyr353 and Phe405 interact ring to ring with the quinolone moiety, showing the best largest van der Waals interactions. Tyr 348, in particularly, makes hydrophobic contacts with the IMMS side chain that originated in atom in position 2.

The IMMS-TLR8 complex has seven to ten hydrophobic residues, but only five of them are common to all IMMS. However, the number of hydrophobic contacts of the same residue may vary depending on the interacting IMMS. Aromatic residues make abundant and different contacts, particularly with the imidazole benzene ring. Thus, the number of van der Waals interactions varies from 32 to 37. Fig 9 shows three examples of hydrophobic interactions of IMMS with their respective interacting residues from the TLR8-dimer binding pocket. Additionally, Table 5 shows the interacting residues and number of hydrophobic interactions.

**Table 5:** Hydrophobic interactions of IMMS in the TLR8 binding pocket.

IMMS	Monomer	Residues	R*	Monomer	Residues	R*	Total R*	Total Inter **
**Hybrid-2**	A	Tyr348, Tyr353, Val378, Phe405,	7	B	Asp545,	2	9	33
		**Phe346, Gly376, Arg429**			**Val573**			
**XG1-236**	A	Tyr348, Tyr353, Val378, Phe405,	6	B	Asp545	1	7	37
		**Gly376, Arg429**						
**DS802**	B	Tyr348, Tyr353, Val378, Phe405,	7	A	Asp545,	2	9	37
		**Phe346, Gly376, Arg429**			**Val573**			
**CL075**	A	Tyr348, Tyr353, Val378, Phe405,	6	B	Asp545	1	7	36
		**Gly351, Ser352**						
**CL097**	B	Tyr348, Tyr353, Val378, PheF405,	7	A	Asp545,	3	10	36
		**Phe346, Gly376, Arg429**			**Gly572, Val573**			
**R848**	B	Tyr348, Tyr353, Val378, Phe405,	6	A	Asp545	1	7	32
		**Phe346, Gly376**						

R* = number of residues.

The top five residues are similar in all cases.

Dissimilar residues are in bold face

Inter** = number of hydrophobic interactions.

### Atomic charges of the IMMS-TLR8 complex

Atomic charges were calculated under unbound and bound conditions for both IMMS and the corresponding atoms of the binding pocket residues. In the unbound condition, the atomic charges of the N atoms 3, 5 and the N11 amino group, which form the polar pharmacophore, are highly negative, especially the N11 atom. In contrast, the two H atoms of the N11 amino group and the adjacent C4 atom are highly positive. As expected, differences in atomic polarization are observed among the six IMMS. The atoms at position 1 exhibit diverse charge values because of the variety of atoms at this position; the same occurs with its contiguous number 2, 9a and 10 atoms. The remaining atoms of the quinoline moiety have either negative (C6 to C9) or positive charges (C3a and C5a). The binding of the IMMS atoms with the TLR8 residues polarizes the quinoline benzene ring atoms as well as the polar pharmacophore atoms, excluding the N11-H atoms, which remain almost unchanged.

**Fig 10:**
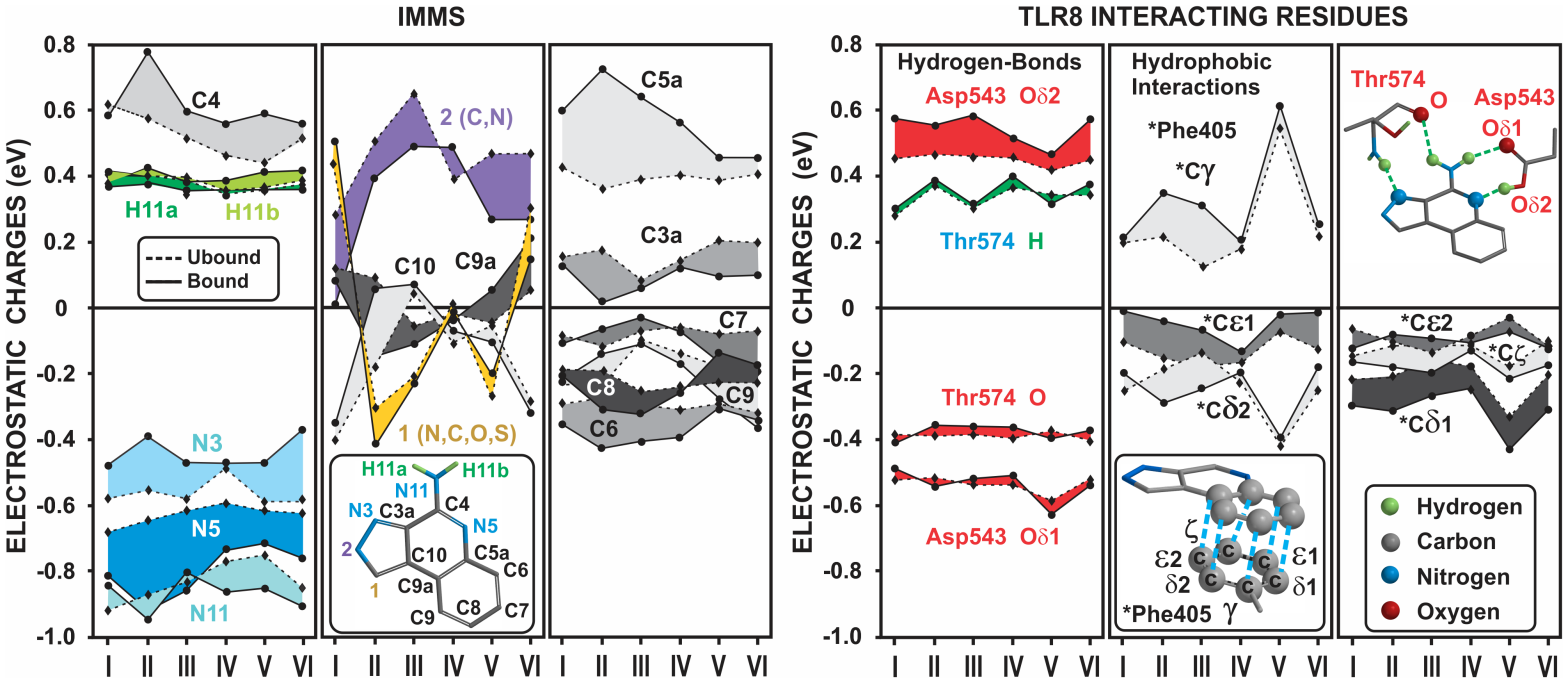
Atomic charge differences among IMMS-TLR8 interacting atoms under bound and unbound conditions. Most of the IMMS atoms (left panel) were polarized by the binding process, increasing and decreasing their charge values. Thus, N3, N5 and N11 polar pharmacophore atoms, along with C4 and C5a, showed a high polarization characteristic. Of note, atom at position 1 (C, N, S, and O) displayed a wide variability in charge but reduced polarization. Carbons 6, 7, 8 and 9 of the benzene ring showed important charge variations in some IMMS. On the TLR8 side (right panel), only atom O-δ2 of Asp545 was modified by the binding to the IMMS N5 atom of the polar pharmacophore. In contrast, the ring atoms of Phe405 were most polarized by hydrophobic interactions in compounds II and III. Unbound and bound charge differences are shown as bands. Molecular and atomic data are shown for the sake of clarity.

In contrast, the interacting carboxylic oxygen atoms (O-δ1 and O-δ2) of Asp543 show different extents of polarization that favors the atom interacting with the N5 atom of the quinoline group. However, the atomic charges of NH, O, and O-δ1 of Thr574 remain almost unchanged. The ring atoms of Phe405 were the most interactive with the quinoline-benzene ring, showing a crowded hydrophobic ring-to-ring interaction. Fig 10 shows the curve tendencies for the groups of atoms in IMMS, and the atoms of the TLR8 residues under both unbound and bound conditions.

### Interaction energy assessment of the IMMS-TLR8 complex

**Fig 11:**
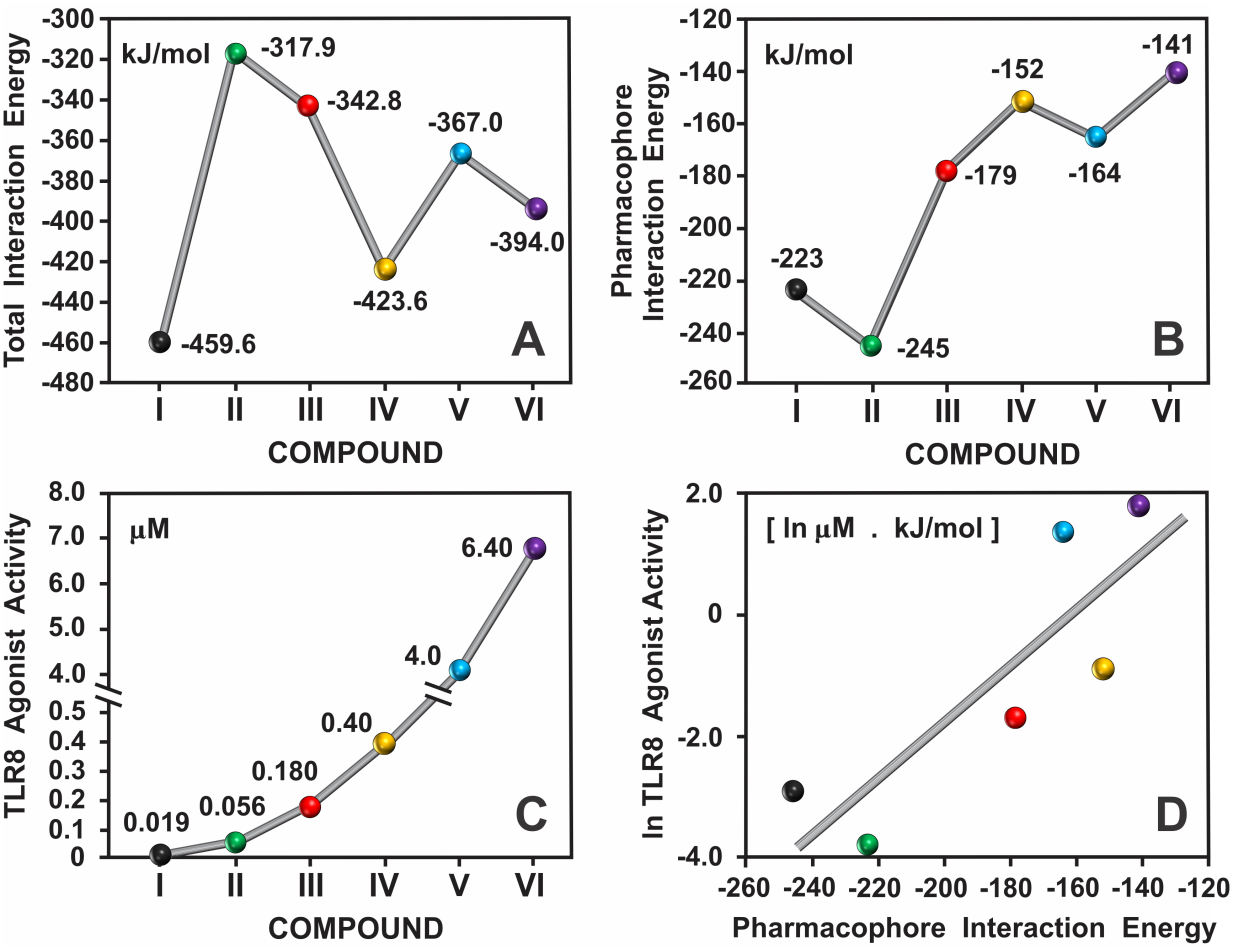
Total and polar pharmacophore interaction energy curves of the IMMS-TLR8 complex. Both interaction energy curves **(A and B)** show nearly opposite trends. However, the third curve **(C)** that shows the values of the TLR8 agonist activity, correlates quite well with the interaction energy of the pharmacophore as observed in **D** were correlation of both curves show values of r = 0.838 and p = 0.037. Curve and coordinates were adjusted to display highest values.

The total interaction energy for each of the six IMMS-TLR8 complexes was calculated, as well as the pharmacophore (polar region) interaction energy. The interaction energy of the IMMS-pharmacophore-TLR8 polar residues was calculated considering the following as a system: C4, N3, N5 and the N11 amino group of IMMS as the pharmacophore and the concurrent Thr574 and Asp543 residues of TLR8, with one fixed water molecule (see Fig 8). Notably, the tendency of the polar pharmacophore interaction energy curve was opposite to that of the total interaction energy. Interestingly, the interaction energy curve elicited by the six complexed polar pharmacophores largely resembles the curve formed by the IMMS experimental pharmacological potency. In fact, the line regression and correlation analysis for both curves showed a linear trend with statistically significant values of r = 0.838 and p = 0.037 (for the pharmacological potency values was used their natural logarithm). Fig 11 shows the two interaction energy curves calculated from the binding pocket of the IMMS-TLR8 complex and the polar pharmacophore-TLR8 -Thr574 and Asp543 interactions. In addition, the potency of the IMMS agonist activity stimulation curve toward IRF-NFκB transcription factors is included for correlation (for values, see Table 1). In panel D is shown the line regression of TLR8 agonist activity (ln values) and pharmacophore interaction energy. The associated data for both interaction energy calculations are shown in Table 6.

**Table 6:** Total interaction energy calculations for the IMMS-TLR8 complex at a distance of 4 Å.

Total Interaction Energy	Hybrid-2 B	XG1-236 B	DS802 A	CL075 B	CL097 A	R848 A
Interaction Energy (kJ/mol)	-459.65	-317.90	-342.81	-423.61	-367.04	-394.02
Total Residues	11	9	11	9	12	9
Total Water	2	1	2	4	2	2
**Pharmacophore Interaction Energy**						
Interaction Energy (kJ/mol)	-223.57	-245.09	-178.53	-151.65	-164.25	-141.45
Total Residues	2	2	2	2	2	2
Total Water	1	1	1	1	1	1
**Interactions**						
Bridged H2O Bonds	5	2	3	6	3	4
Hydrogen Bonds	4	4	4	4	4	4
Hydrophobic Interactions	33	37	37	36	36	32
Total Interactions	42	43	44	46	43	40

## Discussion

The chemical structure of the studied IMMS is quite similar, showing only few differences in the side chains attached to atoms at position 1 and 2 of the imidazol ring. Likewise, atom at position 1 also differs in some cases, showing the following trends for pharmacological effectivity as follows: C<O<S<N-H. The side chain at position 2 is always present and seems to be quite important in compounds I and VI, differing by one atom but exhibiting the highest and lowest pharmacological potency, respectively [13, 24]. Notably, the highest pharmacological potency of compound I correlates quite well with its lower total and polar pharmacophore interaction energy values. A comparison of the binding interactions between compounds I and VI shows that two more residues participate in the binding pocket, two bridge the water bonds and two more atomic interactions favor compound I. These subtle differences account for better interaction energy and increase the pharmacological effectiveness of compound I. Similarly, compounds II and III have different chemical structure only in atom at position 1 with an analogous side chain, which changes their interaction energy and pharmacological potency. Thus, varying the number of fixed water molecules, bridged hydrogen bonds, and number of van der Waals interactions account for the pharmacological effectiveness. Likewise, the low effectiveness of compound V may be due to the ethoxy methyl side chain, which is similar to that of compound VI [24].

Interestingly some physicochemical properties correlated to a large extent; for example, the total energy pattern of the IMMS molecules resembles the total interaction energy curve. Both curves exhibit lower values for compounds I, IV and VI. Additionally, the interaction energy curve emphasize that the binding pocket environment differs for each IMMS-TLR8 complex. Likewise, both the solvation energy and electronegativity curves mimic the polar pharmacophore interaction energy curve, suggesting that the properties are directly associated with that pharmacophoric activity.

In contrast, the frontier orbital (HOMO and LUMO) energy showed an inverse tendency compared with the polar pharmacophore energy. The polar surface area correlates quite well with the polar pharmacophore area excluding compound CL075 (IV), which has a sulfur atom at position 1[15]. In fact, because of its physicochemical properties, the S atom modifies the overall properties of compound IV. Notably, the atoms of the polar pharmacophore were the most polarized: N3, N5 and N11, followed by C5a and C4 to a lesser extent, reinforcing the importance of the polar pharmacophore. Regarding the residues in the binding pocket, only a few carbon atoms of Phe405 and one oxygen atom of Asp843 were slightly polarized. Nevertheless, those residues appear to be essential to activate NF-κB [15].

According to our analysis of the IMMS mechanism of action, the interaction energy (binding) of the polar pharmacophore is the main issue related to the pharmacological potency in the six studied IMMS-TLR8 crystals. The polar pharmacophore region is somewhat different from the four region model proposed by Musmuca *et al*. [26] for the IMMS molecule and related immunomodulators. In an interesting 3-D QSAR analysis of 156 interferon-inducing agents, those authors described a pharmacophoric model with four regions, including the adenine moiety with two regions: the HA region and a polarized area in which those authors suggested the presence of hydrogen bonds and polar interactions. Both regions are concurrent with the N3, N5 and N11 amino group of the IMMS, which we are denoting as the polar pharmacophore, that also includes the hydrogen bonds network with a fixed water molecule and the two polar residues of the TLR8 binding pocket. The other two pharmacophoric regions proposed by Musmuca *et al*. [26] are a fillable steric pocket and a hydrophobic area.

The two studied IMMS antagonists, compounds 15 and 16 [16], have important molecular modifications, particularly in the polar pharmacophoric region. Thus, both molecules contain the N3 atom covalently bound to a large 2-methyl-propan-2-ol side chain. Additionally, compound 16, the most effective antagonist, lacks the pharmacophoric N11 amino group. These molecular changes, which block or delete the polar region, clearly show that this region is essential to activate the immune response through the IMMS-TLR complex. Therefore, the molecular mechanism of the antagonist is likely due to the absence of the fixed water molecule, the absence of the hydrogen bonds network and null or a low contribution of residues Asp545 and Thr574. However, both antagonist compounds are able to occupy the binding pocket and interact, likely by increased van der Waals forces by their two side chains. Additionally, the two antagonists have the DM vector directed toward the hydrophobic region; the compound 15 vector is slightly shorter, and both possess a high solvation energy, implying low solvation. Those features apparently favor hydrophobicity and, likely because of a distinct electronic environment, results in the antagonist effect. In contrast, compounds I and VI have the DM vector toward the polar region, decreasing the solvation energy and, therefore, increasing the solvation of the molecule. In this regard, compound I has the lowest solvation energy and the largest DM pointing toward N5. Likewise, a comparison of compounds II and III shows that compound II has the lowest solvation energy and a higher DM, whereas compound III has the shortest DM. All these data correlate quite well with the pharmacological effectiveness of these compounds.

Analysis of the pharmacological potency of IMMS reveals almost the same trend for the activation of both TLR7 and TLR8. However, compounds I, V and VI are clearly more capable of stimulating TLR7 at lower doses. In contrast, TLR8 responds better to compounds II, III and IV. This difference occurs regardless of the close phylogenetic relationship of TLR7 and TLR8 to the same TLR subfamily [27], showing a high level of sequence homology [28, 29]. In fact, a theoretical model predicts different residues for the binding site of TLR7 with respect to TLR8 [30]. Thus, the differences in IMMS potency may be related to the intrinsic structural disparities between the TLR7 and TLR8 binding pocket, which yield functional differences for both receptors, as previously proposed by Gorden *et al*. [23]. Additionally, because of their different hydrophobic side chains, IMMS may possess TLR selectivity similar to that reported for R837 (imiquimod), which specifically activates TLR7 [15].

The small ligands IMMS activate both TLR7 and TLR8 similarly to the natural pathogens to yield a cascade of biochemical events that initially stimulate several type of cells, such as monocytes and NK/NKT, T, B, mast and tumor cells. Most of these cells in turn undergo migration, proliferation and apoptosis, along with the production of considerable amounts of interferons, interleukins and tumor necrosis factors, among others [31]. The biochemical reactions of the immune system elicited by IMMS have been confirmed to be effective against viral infections [32]. In fact, TLR7 and TLR8 sense single-stranded viral RNA [33]. The pleiotropic characteristics of the IMMS that trigger the immune response also play an important role in improving cancer therapy, regarding this, see for instance [31] and the important review of Schön and Schön [34]. Besides, IMMS show an angiogenesis inhibitor effect [35] and likely act as vaccine adjuvants [36]. According to our data, all of these pharmacological effects may be correlated, to a large extent, to the regional interaction energy of IMMS. We further validate the usefulness of the crystal analysis as a tool to unveil new data that can be used to improve the design of the IMMS molecule to produce better immunostimulant responses by lowering toxicity and enhancing effectiveness.

## Conclusions

The present results show that the application of quantum mechanics theory and computational chemistry methods to analyze the crystal structure of TLR8 interacting with different small synthetic IMMS, focusing particularly on the binding site of the receptor and the IMMS atomic interactions, enabled elucidation of the molecular properties and calculation of the interaction energy of the IMMS-TLR8 complex, which correlates quite well with the immunopharmacological activity.

The IMMS molecules clearly bears a defined polar region that acts as a regional pharmacophore, which seems to have variable selectivity for TLR7 and TLR8. This finding might facilitate the rational design and development of new agonist and antagonist IMMS that function specifically or selectively in the TLR system or toward other receptors capable of detecting pathogen-associated molecular patterns (PAMP), improving the therapeutic modulation of natural immunity and therapeutic efficiency toward infectious and neoplastic diseases.
